# New Synthetic Cannabinoids Metabolism and Strategies to Best Identify Optimal Marker Metabolites

**DOI:** 10.3389/fchem.2019.00109

**Published:** 2019-03-04

**Authors:** Xingxing Diao, Marilyn A. Huestis

**Affiliations:** ^1^Shanghai Institute of Materia Medica, Chinese Academy of Sciences, Shanghai, China; ^2^The Lambert Center for the Study of Medicinal Cannabis and Hemp, Institute for Emerging Health Professions, Thomas Jefferson University, Philadelphia, PA, United States

**Keywords:** novel psychoactive substances, NPS, synthetic cannabinoid, SC, metabolism, urinary metabolites, hepatocyte incubation

## Abstract

Synthetic cannabinoids (SCs) were initially developed as pharmacological tools to probe the endocannabinoid system and as novel pharmacotherapies, but are now highly abused. This is a serious public health and social problem throughout the world and it is highly challenging to identify which SC was consumed by the drug abusers, a necessary step to tie adverse health effects to the new drug's toxicity. Two intrinsic properties complicate SC identification, their often rapid and extensive metabolism, and their generally high potency relative to the natural psychoactive Δ^9^-tetrahydrocannabinol in cannabis. Additional challenges are the lack of reference standards for the major urinary metabolites needed for forensic verification, and the sometimes differing illicit and licit status and, in some cases, identical metabolites produced by closely related SC pairs, i.e., JWH-018/AM-2201, THJ-018/THJ-2201, and BB-22/MDMB-CHMICA/ADB-CHMICA. We review current SC prevalence, establish the necessity for SC metabolism investigation and contrast the advantages and disadvantages of multiple metabolic approaches. The human hepatocyte incubation model for determining a new SC's metabolism is highly recommended after comparison to human liver microsomes incubation, *in silico* prediction, rat *in vivo*, zebrafish, and fungus *Cunninghamella elegans* models. We evaluate SC metabolic patterns, and devise a practical strategy to select optimal urinary marker metabolites for SCs. New SCs are incubated first with human hepatocytes and major metabolites are then identified by high-resolution mass spectrometry. Although initially difficult to obtain, authentic human urine samples following the specified SC exposure are hydrolyzed and analyzed by high-resolution mass spectrometry to verify identified major metabolites. Since some SCs produce the same major urinary metabolites, documentation of the specific SC consumed may require identification of the SC parent itself in either blood or oral fluid. An encouraging trend is the recent reduction in the number of new SC introduced per year. With global collaboration and communication, we can improve education of the public about the toxicity of new SC and our response to their introduction.

## Introduction

The endogenous cannabinoid system includes neurotransmitters or endogenous cannabinoids, receptors, cannabinoid receptor 1 (CB_1_, mainly expressed in brain) and CB_2_ (particularly abundant in immune tissues), and synthetic and degradation pathways (Pertwee, 2012; Le Boisselier et al., 2017). Cannabinoid pharmacology continues to expand with the identification of other signaling pathways including the TRP receptors and gated ion channels. Cannabinoid receptor ligands are also found in phytocannabinoids, particularly Δ^9^-tetrahydrocannabinol (THC), the primary psychoactive constituent produced in the cannabis plant (Gurney et al., 2014; Carlier et al., 2017a). Cannabis is the most widely used illicit drug globally, with the World Health Organization estimating that in 2013, 181.8 million people aged 15–64 used cannabis for nonmedical purposes (World Health Organization, 2016). The United Nations Office on Drugs and Crime World Drug Report 2015 indicated that SCs represented 39% of all new psychoactive substances (United Nations, 2016). From 2014 to 2015, 177 SCs were reported to the United Nations Office on Drugs and Crime, with reports from 58 countries and territories.

**Figure 1:**
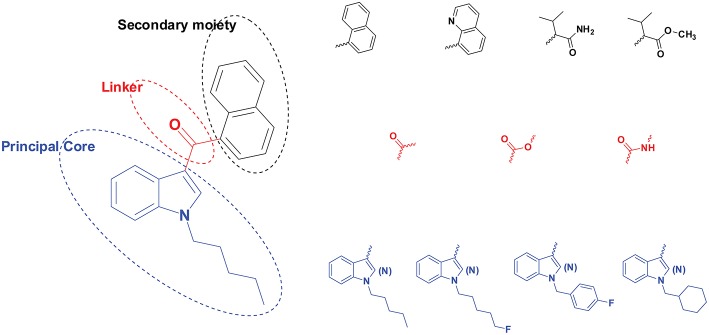
Structural scheme of synthetic cannabinoids (SCs) with different principal cores (blue), linkers (red), and secondary moieties (black). A few examples are provided for each moiety's diverse substructures.

Initially, synthetic cannabinoids (SCs) were developed as pharmacological probes to explore the endogenous cannabinoid system with potential treatment for inflammatory diseases and cancer pain (Pertwee, 2006; Castaneto et al., 2014), but such endeavors failed to date, with no SC progressing to clinical use. Typical SC structures are shown in Figure 1. Synthetic THC is an approved pharmacotherapy for the reduction of nausea and vomiting following chemotherapy and to stimulate hunger in HIV AIDS wasting disease. In June 2018, the US Food and Drug Administration approved the first cannabinoid plant extract (Epidiolex®) for the treatment of seizures associated with two rare and severe forms of epilepsy, Dravet's and Lennox-Gastaut seizure syndromes (US Food Drug Administration, 2018). Epidiolex has a high CBD content and < 0.3% THC.

Many SCs are potent CB_1_ and/or CB_2_ agonists and elicit cannabimimetic effects similar to THC. SCs were first identified as recreational drugs of abuse in 2008 in Europe and Japan (Elsohly et al., 2014; Kemp et al., 2016). SC are usually synthesized by clandestine laboratories mainly in Asia, sold over the internet and labeled “not for human consumption” (Figure 1). Initially, SC were sprayed on dried plant material, but currently, small bottles of solubilized SC are shipped to help avoid custom detection, and the end user smokes or vapes the product (Spice Addiction Support Organization, 2019).

Due to the important role of the endogenous cannabinoid system in human health and behavior, the acute and chronic effects of SC exposure are the primary concerns among the scientific community, as described in recent reviews of their adverse neurological, psychiatric, cardiorespiratory, and gastrointestinal effects (Castaneto et al., 2014; Panlilio et al., 2015; Cooper, 2016; Logan et al., 2017). Most common clinical toxicities were not life-threatening, such as tachycardia, agitation, drowsiness, vomiting/nausea, hallucinations, confusion, hypertension, chest pain, dizziness/vertigo (Tait et al., 2016); however, more severe outcomes, including death occur (Elliott et al., 2015; Waugh et al., 2016). A notorious outbreak associated with SCs occurred in the State of Mississippi in April and May 2015. This public health emergency claimed 17 lives and involved 1,243 emergency room visits (Mississippi State Department of Health, 2015; Kemp et al., 2016). Ingestion of the toxic SC, later identified as MAB-CHMINACA, resulted in hospitalization of more than 10% of the patients into intensive care units. The Australian National Household's first survey reported that among Australians over 14 years old, 1.2% used SCs in the previous year, and 0.4% used other NPS (Australian Institute of Health Welfare, 2014). A total of 858 SC were scheduled in Japan as narcotics or designated substances as of April 2015 (Uchiyama et al., 2015).

Confirmation of SC identity is important to tie the adverse events to the specific toxic compound and because different SC analogs may have different scheduling status. For instance, some SCs (such as THJ-018 and THJ-2201) share similar urinary marker metabolites; THJ-2201 was scheduled in the United States while THJ-018 was not (Drug Enforcement Administration, 2017). Rarely do drug-abusers know which SC or SCs they consumed, and severe potential drug-drug interactions with other abused illegal drugs or therapeutics can occur (Chimalakonda et al., 2012).

## Why do we Study SC Metabolism?

Constantly emerging SCs pose a significant challenge for forensic laboratories performing drugs-of-abuse testing, as initially SCs are not incorporated into existing targeted screening methods. One of the greatest current challenges in forensic toxicology is the large number of novel psychoactive substances available, and the difficulty in identifying the best analytical targets to detect their abuse. All previously investigated SCs were extensively metabolized, with little to no unchanged parent drug found in human urine (Scheidweiler et al., 2015; Cannaert et al., 2016; Diao et al., 2016b; Carlier et al., 2017b). Urine is the most common matrix for drug testing because of its non-invasive collection, adequate sample, higher drug concentrations and longer detection window than either blood or oral fluid (Hutter et al., 2018). Generally, phase I metabolites are the best SC marker metabolites to document intake because they have higher mass spectrometry responses and are more stable than phase II metabolites over time. Forensic urine samples are usually hydrolyzed by β-glucuronidase prior to mass spectrometry analysis, increasing sensitivity by measuring free and glucuronidated moieties. Of note, some hydroxylated urinary metabolites are even more toxic than the parent SC themselves; JWH-018 major metabolites, 4′-OH-JWH-018 and 5′-OH-JWH-018, and AM-2201 metabolite, 4′-OH-AM-2201, remained full agonists in nanomolar concentrations (Chimalakonda et al., 2012). Therefore, metabolism studies on novel emerging SC are essential.

The best approaches for investigating SC metabolism are in humans. Unfortunately, controlled SC administration studies are severely restricted due to ethical limitations. The first tenet for controlled human administration studies is do no harm, and the lack of acute and chronic drug toxicity data limit our ability to conduct such studies. It also makes no sense to routinely perform such clinical trials for each new emerging SC. Other approaches are available to identify and confirm human urinary SC marker metabolites.

## Models to Study SC Metabolism

The biotransformation of xenobiotics converts drugs into more water soluble metabolites to achieve better elimination from the human body (Costa et al., 2014). Although drug metabolism occurs in the lungs, kidneys, intestine, heart and blood, the most important organ for drug metabolism process is the liver, with its many hepatic enzymes, especially those of the cytochrome P450 (CYP) family (Brandon et al., 2003; Xie et al., 2013; Diao et al., 2015; Zhu et al., 2016).

*In vitro* models provide useful tools to assess human drug metabolism according to their ability to reproduce human biotransformations. In this review, the most common and well-established *in vitro* metabolism models, human hepatocytes and human liver microsomes (HLM), are compared in light of their advantages and disadvantages; other approaches such as *in silico* software prediction, rat *in vivo*, zebrafish incubation, and fungus *Cunninghamella elegans* (*C. elegans*) models were also reviewed.

### Human Hepatocyte Incubation

Human hepatocyte incubation is an excellent *in vitro* system for drug biotransformation research due to its ability to reflect metabolism in the intact human liver. Human hepatocytes are isolated living cells containing the complete repertoire of phase I and phase II drug metabolizing enzymes, necessary cofactors, uptake and efflux drug transporters, and drug binding proteins (Diao and Huestis, 2017).

At the National Institutes of Health/National Institute on Drug Abuse, we established a strong collaboration with the United States Drug Enforcement Administration (DEA) to identify optimal marker metabolites of new SC. When DEA seizures of a particularly toxic new SC they would purify and provide us with the SC. We first determined the SC's half-life by quantifying the disappearance of the parent compound during incubation with HLM, in order to best design the SC human hepatocyte incubation experiment. By far, the most challenging aspect was the high-resolution mass spectrometry (HR-MS) analysis to identify the full spectrum of metabolites, and those metabolites that best differentiated the SC from its closest analogs. We also attempted to obtain authentic human urine samples following specific SC ingestion through international collaborations, enabling comparison of *in vitro* and *in vivo* metabolites and clarifying the targets for SCs drug testing.

**Figure 2:**
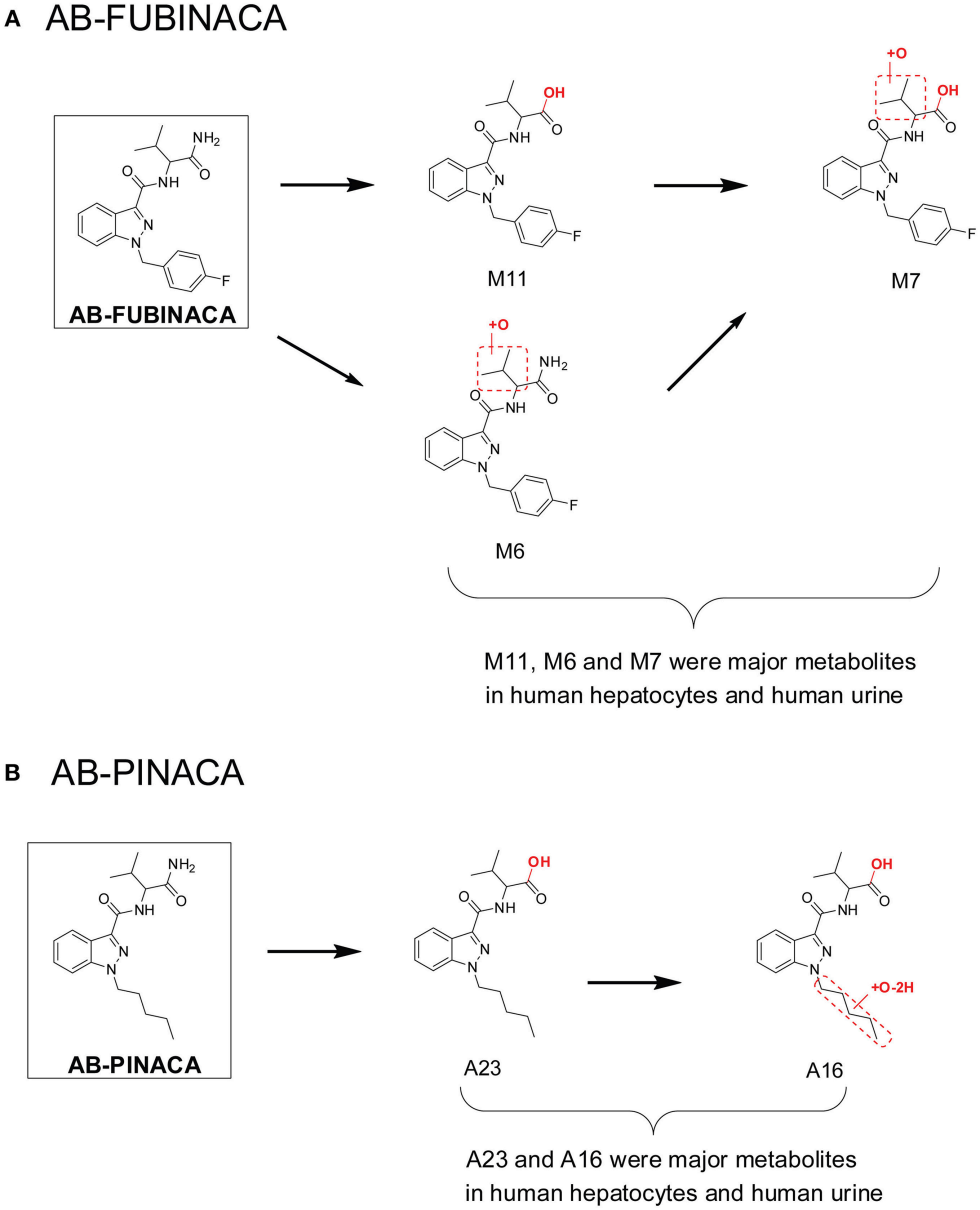
Major metabolites of AB-FUBINACA **(A)** and AB-PINACA **(B)** following human hepatocytes incubation and in human urine samples after suspected AB-FUBINACA and AB-PINACA intake. All metabolite nomenclatures are from the original manuscripts.

This workflow was successful in predicting major urinary metabolites of many SCs, including AB-PINACA/5F-AB-PINACA (Wohlfarth et al., 2015), AB-FUBINACA (Castaneto et al., 2015), FDU-PB-22/FUB-PB-22 (Diao et al., 2016a), and NM-2201 (Diao et al., 2017b) etc. For AB-FUBINACA, the prominent metabolite following human hepatocyte incubation was amide hydrolysis product M11 (Figure 2A). Consistently, M11 was also the most abundant metabolite in human urine after β-glucuronidase hydrolysis. Besides M11, M6 (aliphatic hydroxylation) and M7 (amide hydrolysis product of M6) were the primary metabolites after β-glucuronidase hydrolysis in both human hepatocyte incubation and in human urine following AB-FUBINACA intake. For, AB-PINACA, metabolites A23 and A16 were the major metabolites in human hepatocyte incubation and in human urine after β-glucuronidase hydrolysis (Figure 2B).

#### Advantages

With modern cryopreservation techniques, high quality isolated human hepatocytes are commercially available, and retain the activity of most phase I and II enzymes (Silva et al., 1999). High quality metabolic data are produced by this well-established and well-characterized *in vitro* model.

#### Disadvantages

Cryopreserved human hepatocytes are much more expensive than HLM, and once a vial is thawed, it should be fully utilized and never refrozen. Storage under liquid nitrogen is required and the viability of the hepatocytes must be checked after thawing. Human hepatocytes account for about 80% of total liver volume; however, other cells, i.e., Kupffer cells, may supply additional required cofactors.

### HLM Incubation

HLM incubation is currently the most popular *in vitro* metabolism model, providing an affordable method to identify target metabolites following CYP and UGT metabolism. The popularity of this *in vitro* model is attributed to its simplicity and widespread availability, and the ability to determine specific metabolizing isozyme(s) by studying their activity in the presence of specific inhibitors (Bickett et al., 1993). HLM are hepatocyte endoplasmic reticulum vesicles prepared by differential centrifugation. HLM contain primarily CYP, UGT and esterase enzymes, accounting for about 95% of clearance mechanisms for the top 200 drugs prescribed in the United States in 2002 (Williams et al., 2004).

**Figure 3:**
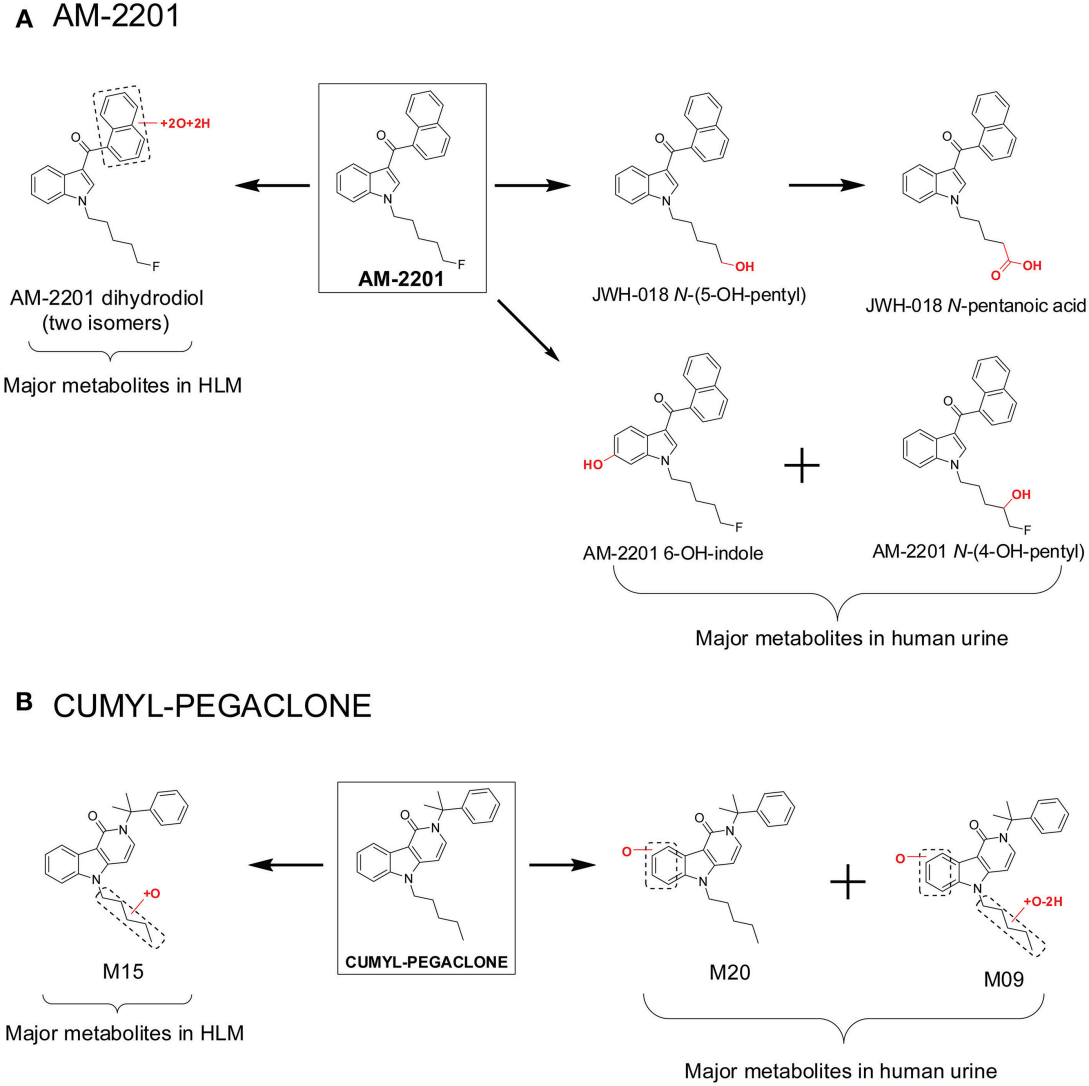
Major metabolites of AM-2201 **(A)** and CUMYL-PEGACLONE **(B)** following human liver microsomes (HLM) incubation and in human urine samples after suspected AM-2201 and CUMYL-PEGACLONE intake.

However, major metabolite discrepancies may occur between those noted in HLM incubations and those found in human urine following SC intake, i.e., in the metabolism of 5F-AKB-48 and AM-2201 (Figure 3A; Diao and Huestis, 2017). The probable reason is that the enzyme responsible for oxidative defluorination, the primary metabolic pathway for a fluoropentyl chain SC, is not located in HLM. In the case of AM-2201, the HLM metabolic profile did not match satisfactorily with metabolites identified in authentic urine specimens. AM-2201 HLM incubation produced *N*-desfluoropentyl, mono-hydroxyl, di-hydroxyl, and the most abundant dihydrodiol metabolite (Figure 3A; Sobolevsky et al., 2012). One researcher self-administered 5 mg AM-2201 to identify the major human urinary metabolites (Hutter et al., 2013). Four major metabolites were identified in the post administration urine samples, JWH-018 *N*-(5-OH-pentyl), JWH-018 *N*-pentanoic acid, AM-2201 6-OH-indole, and AM-2201 *N*-(4-OH-pentyl). The highest concentrations were for JWH-018 *N*-pentanoic acid and JWH-018 *N*-(5-OH-pentyl); however, AM-2201 shared major metabolites with JWH-018, i.e., JWH-018 *N*-(5-OH-pentyl) and JWH-018 *N*-pentanoic acid. Thus, it is challenging to differentiate AM-2201 from JWH-018 intake based on the detection of these two metabolites in urine specimens. To distinguish AM-2201 intake from JWH-018, detection of AM-2201 6-OH-indole and AM-2201 *N*-(4-OH-pentyl) is essential.

Recently, Mogler et al. also reported discrepancies in the metabolism of SC CUMYL-PEGACLONE between HLM and human urine samples (Mogler et al., 2018). Following HLM incubation, M15, a mono-hydroxylation metabolite on the pentyl chain, was found in the highest concentration, and no parent, CUMYL-PEGACLONE was detected in any of the human urine samples (*n* = 30). Twenty-two different phase I CUMYL-PEGACLONE metabolites were detected in human urine. Metabolic pathways included mono-hydroxylation, di-hydroxylation, dehydrogenation, N-dealkylation, β-oxidation (pentyl side chain to a propionic acid metabolite), carbonyl formation at the pentyl side chain, and combinations of these biotransformations. The most abundant two metabolites were identified as M20 and M09 (Figure 3B); M20 was a metabolite with mono-hydroxylation on the γ-carbolinone core and M09 was a further pentyl chain carbonylated metabolite from M20. The authors proposed M20 and M09 as sensitive and specific urinary markers to prove intake of CUMYL-PEGACLONE. The metabolism of CUMYL-PEGACLONE was unexpectedly different from previous cumyl-derivatives (Kevin et al., 2017; Özt"urk et al., 2018; Staeheli et al., 2018), which were mainly hydroxylated on the pentyl side chain, whereas CUMYL-PEGACLONE was mainly hydroxylated on the γ-carbolinone core.

#### Advantages

The major advantages of the HLM *in vitro* model are its simplicity, low cost and well established record in drug biotransformation research. These advantages facilitated recent investigation on the structure-metabolism relationships of valine and tert-leucine-derived SCs (Franz et al., 2019). Also, the specific enzyme producing a metabolite can be identified with a simple HLM system and specific inhibitors.

#### Disadvantages

The primary disadvantage is that HLM results cannot quantitatively estimate *in vivo* human biotransformation, because CYPs and UGTs are enriched in HLM and there is a lack of competition with other enzymes. Also, drugs are exposed directly to the metabolizing enzymes in HLM, without the requirement to penetrate through cell membranes, as for metabolism in authentic hepatocytes. This results in higher biotransformation rates in HLM compared to the human *in vivo* situation, but also compared to primary hepatocytes (Sidelmann et al., 1996). Additionally, the absence of other enzymes (e.g., Aldehyde oxidase [AOX], N-acetyltransferase [NAT], Glutathione S-transferases [GST], and Sulfotransferase [SULT]) and cytosolic cofactors may fail to produce metabolites formed in intact hepatocytes (Diao et al., 2014). Unlike with human hepatocyte incubations, scientists must determine which co-factors to supplement in HLM incubations. This requires extensive drug metabolism knowledge, especially when the metabolic pathway and enzymes involved are unknown. In addition, co-factors are expensive, although commercially available.

The use of HLM incubations rather than hepatocyte incubations in preclinical toxicology studies was the cause behind the termination of c-Met inhibitor SGX-523 development. The primary SGX-523 metabolic pathway in humans is oxidation by aldehyde oxidase (AOX), yielding 2-quinolinone-SGX523. The much lower solubility of 2-quinolinone-SGX-523 in urine vs. SGX-523 is considered the major reason for renal toxicity. AOX is located in the liver cytosol rather than in liver microsomes. AOX expression is species specific, with presence in humans and monkeys, and little in mouse, rat and dog. During the early drug discovery phase, species comparison studies were performed in liver microsomes, with the results misleading the team to use rat and dog as the toxicology model animals. Since there was little AOX enzyme expression in rat and dog, no toxic metabolites were produced and the potential for human toxicity was overlooked.

### *In silico* Prediction

In pharmaceutical industry, early prediction of possible toxic metabolites is important to preclinical and clinical decision-making (Afzelius et al., 2007; T'Jollyn et al., 2011). Identification of possible toxic metabolites in the drug discovery stage enables earlier decisions on elimination of drug candidates from further development. Adams et al utilized metabolism prediction software to suggest that the urinary AMB-FUBINACA de-esterified acid metabolite could identify AMB-FUBINACA intake in a mass intoxication outbreak (Adams et al., 2017).

In 2011, T'jollyn et al. evaluated Meteor (Lhasa Ltd., Leeds, UK), MetaSite (Molecular Discovery Ltd., Middlesex, UK), and StarDrop (Optibrium Ltd., Cambridge, UK) software drug metabolism tools (T'Jollyn et al., 2011). Meteor is a rule-based (empirical) software tool (Langowski and Long, 2002). MetaSite is an automated docking model with a reactivity correction considering the reactivity components of an atom related to heme that is designed to predict phase I CYP450 metabolism (Cruciani et al., 2005). StarDrop uses a quantum mechanical approach for the prediction of the relative involvement of CYP3A4, 2D6, and 2C9 of the query compound (Earnshaw, 2010). Its mechanism is based on calculation of the energy barrier to electron removal, considered to be the rate-limiting step in product formation. The authors evaluating the state-of-the-art metabolite prediction software concluded that it has many advantageous features but needs refinement to obtain acceptable prediction profiles. Synergistic use of different software packages could prove useful. However, it is not practical for forensic laboratories to purchase expensive *in silico* software licenses.

**Figure 4:**
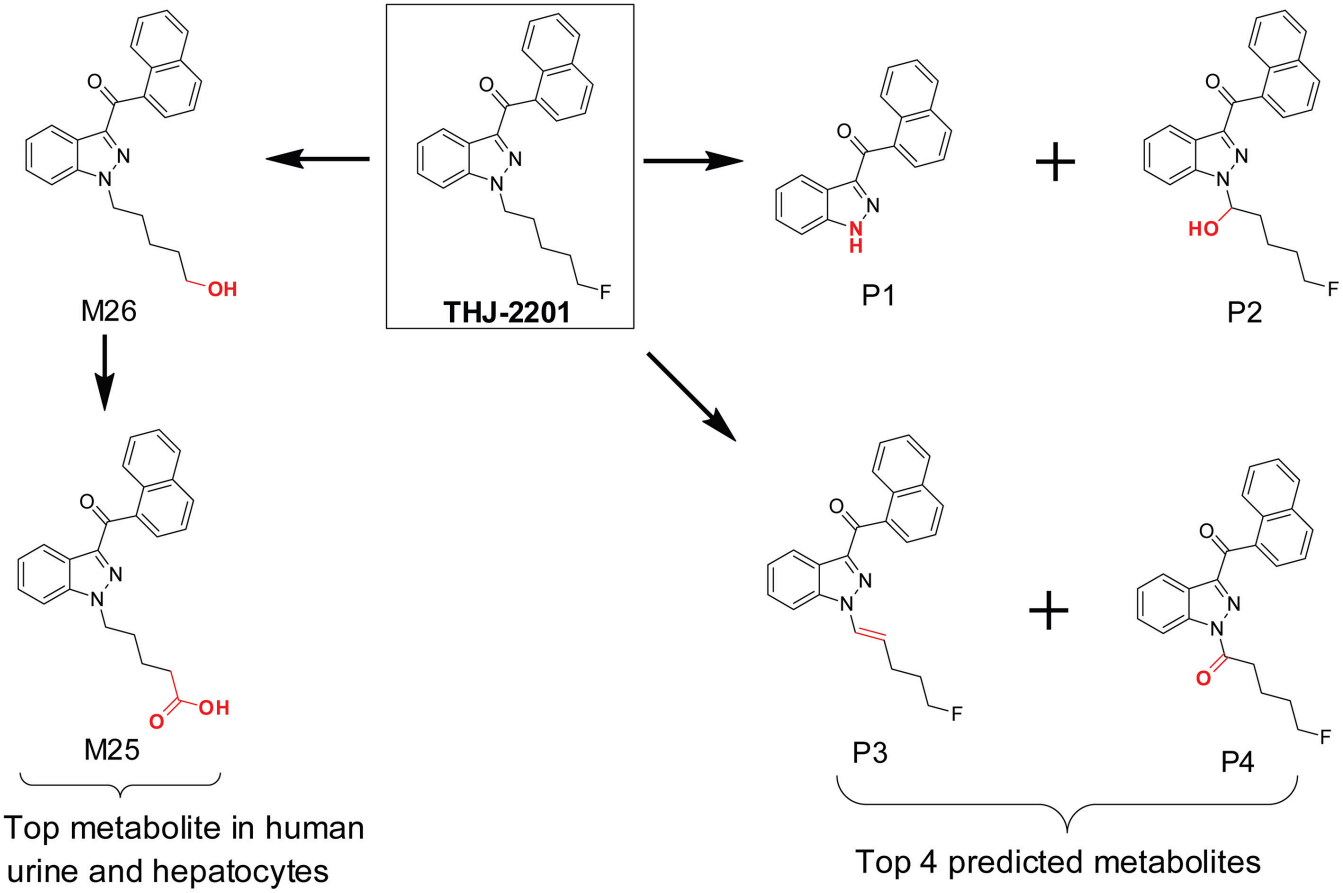
*In silico* predicted THJ-2201 metabolites vs. its major metabolites observed after hepatocytes incubation and in human urine after THJ-2201 intake.

In our previous investigation of SC THJ-2201 metabolism, we first utilized the MetaSite *in silico* prediction software prior to hepatocyte incubation and HR-MS analysis. The software predicted 8 first-generation and 7 second-generation metabolites (Diao et al., 2016c). The top predicted metabolites were *N*-depentyl-THJ-018, 1′-OH-THJ-2201, 1′-carbonyl-THJ-2201, and pent-1′-enyl-THJ-2201 (Figure 4). However, these top predicted metabolites were inconsistent with the scenario in human hepatocyte incubation and human urine. After incubating THJ-2201 with human hepatocytes, 27 metabolites were generated, with THJ-018 pentanoic acid (F25) and 5′-OH-THJ-018 (F26) as the most abundant metabolites. F26 was produced by oxidative defluorination of THJ-2201, with further oxidation to F25. In a SC screening method, THJ-018 *N*-pentanoic acid was the target marker metabolite for THJ-2201 intake (Gundersen et al., 2019).

MetaSite primarily focuses on CYP450 mediated metabolism and does not simulate reactions mediated by non-CYP450 oxidases, such as aldehyde oxidase. The performance of MetaSite for THJ-2201 metabolism most likely missed the oxidative defluorination reaction because it was not catalyzed by CYP450.

#### Advantages

The major advantages of *in silico* software prediction are its simplicity and rapidity. *In silico* prediction assists metabolite identification without requiring a reference standard, incubation or HR-MS.

#### Disadvantages

Some metabolism software does not include all drug metabolizing enzymes in the simulation model, thus missing some metabolite pathways. The *in silico* prediction model may be effective for some drugs and SCs, but are not accurate for all.

### Rat *in vivo* Model

**Figure 5:**
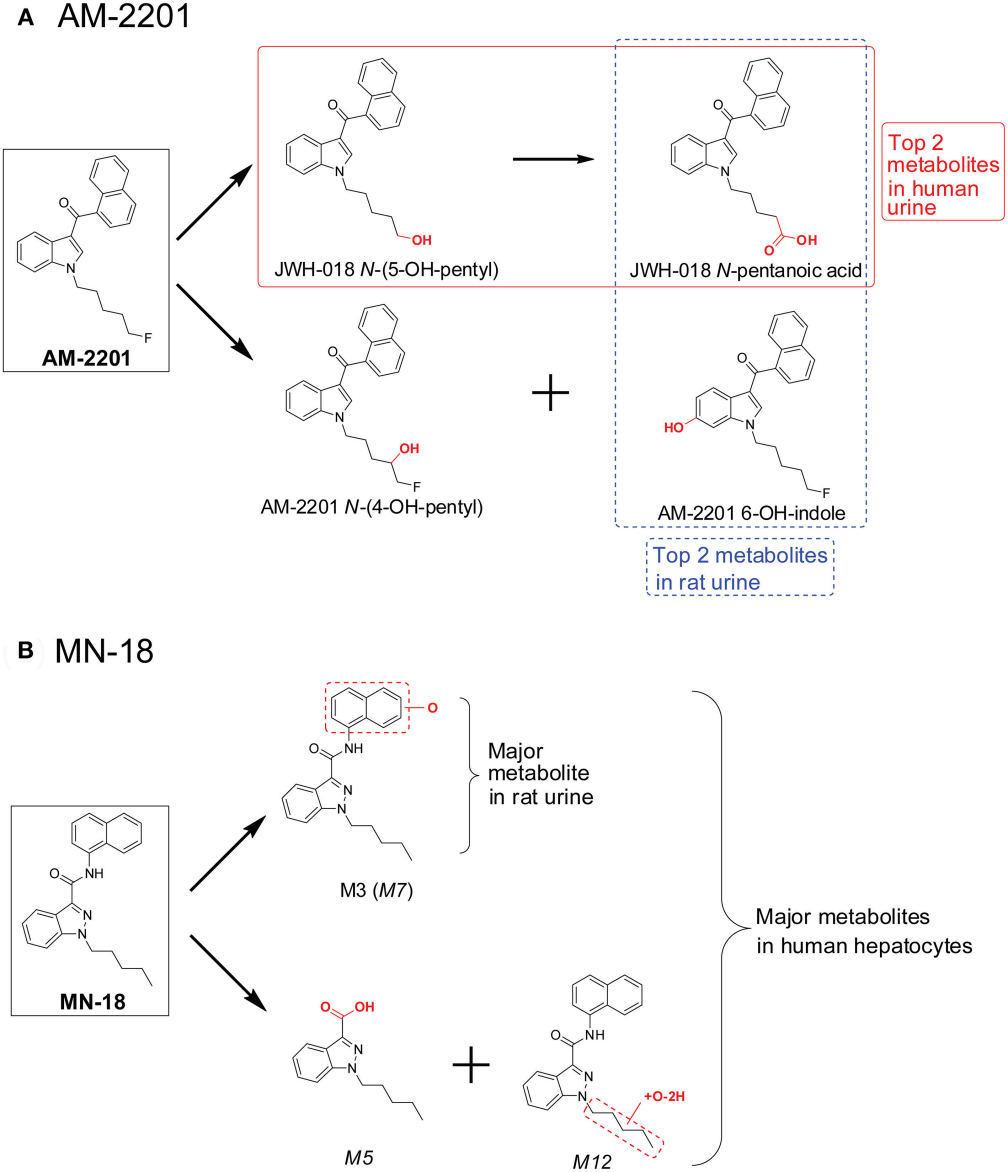
Major AM-2201 metabolites **(A)** in rat urine vs. human urine and **(B)** MN-18 metabolites in rat urine vs. those after human hepatocytes incubation.

Clinical studies on SC effects are hampered by the lack of preclinical toxicology data. It also is impractical to conduct clinical trials on each new SC due to the constant introduction of novel SC into the illegal drug market. Thus, many researchers study SC pharmacodynamics and pharmacokinetics *in vivo* animal models. AM-2201 metabolism in rats has similarities and differences from that in humans (Jang et al., 2014). Predominant AM-2001 metabolites after hydrolysis of rat urine with β-glucuronidase were JWH-018 *N*-pentanoic acid and AM-2201 6-OH-indole (Figure 5A). Two additional metabolites, JWH-018 *N*-(5-OH-pentyl) and AM-2201 *N*-(4-OH-pentyl), were detected in lower abundance in rat urine. These four metabolites also were the top four metabolites in human urine after self-administration of 5 mg AM-2201 (Hutter et al., 2013). However, the relative abundance of these 4 metabolites in human urine was quite different from that in rat urine. In human urine, JWH-018 *N*-pentanoic acid and JWH-018 *N*-(5-OH-pentyl) were the most abundant metabolites (Figure 5A), while AM-2201 *N*-(4-OH-pentyl) and AM-2201 6-OH-indole were observed in lower concentrations.

Recently, Kevin et al. investigated MN-18 metabolism (Kevin et al., 2018) in rat after intraperitoneal administration of 3 mg/mL MN-18. Only two metabolites were identified in rat urine, the hydroxylated metabolite M3 and its glucuronide M10 (Figure 5B). However, following human hepatocyte incubation with MN-18, 13 metabolites were observed (Figure 5B), with the top 3 metabolites 1-pentyl-1H-indazole-3-carboxylic acid (*M5*), naphthalene hydroxylated MN-18 (*M7*), and pentyl-carbonylated MN-18 (*M12*) (Diao et al., 2017a). To reduce confusion, metabolite names were directly taken from the original literature; if the same nomenclature occurred in both literatures, the font of the metabolites from the latter one is in italicized.

#### Advantages

It is much easier and inexpensive to perform an *in vivo* rat or mouse metabolism study than a controlled human drug administration study. The collection of animal plasma and urine samples is relatively easy. The rat model also has the advantage of producing metabolite reference standards. If a major human metabolite is present in rat urine, sufficient urine may be collected to isolate and characterize metabolites and produce reference SC metabolite standards. Another significant advantage of employing a rodent model is that it is possible to observe and measure animal behavior and physiology while conducting the SC metabolism study. In addition, the rodent model may detect acute SC toxicity.

#### Disadvantages

Species differences in metabolism exist, as noted for the AOX enzyme in the metabolism of SGX-523. AOX activity was high in humans and monkeys, but almost absent in rat and dog. Some researchers tried to study SC metabolism in the chimeric mouse with humanized liver (De Brabanter et al., 2013). However, the effectiveness of this model remains unclear because it was not compared to major metabolites in human urine samples. Even if the humanized mouse model is effective, the data quality produced does not justify the high cost; additionally, its limited commercial availability prevents its usage in routine metabolism research.

### Zebrafish Model

The zebrafish (*Danio rerio*) was initially introduced by Streisiger as an animal model in genetic studies in the early 1980s; the zebrafish is a small teleost (3–4 cm) typically from sweet waters (Streisinger et al., 1981). Some important advantages are associated with zebrafish for research, such as small size, easy maintenance, low cost of breeding, and high reproductive rate. Zebrafish is emerging as a predictive vertebrate animal model for *in vivo* assessment of drug efficacy, toxicity, and safety. Interestingly, some studies evaluated the ability of zebrafish larvae at different stages of ripening to generate xenobiotic metabolites (Alderton et al., 2010; Chng et al., 2012).

**Figure 6:**
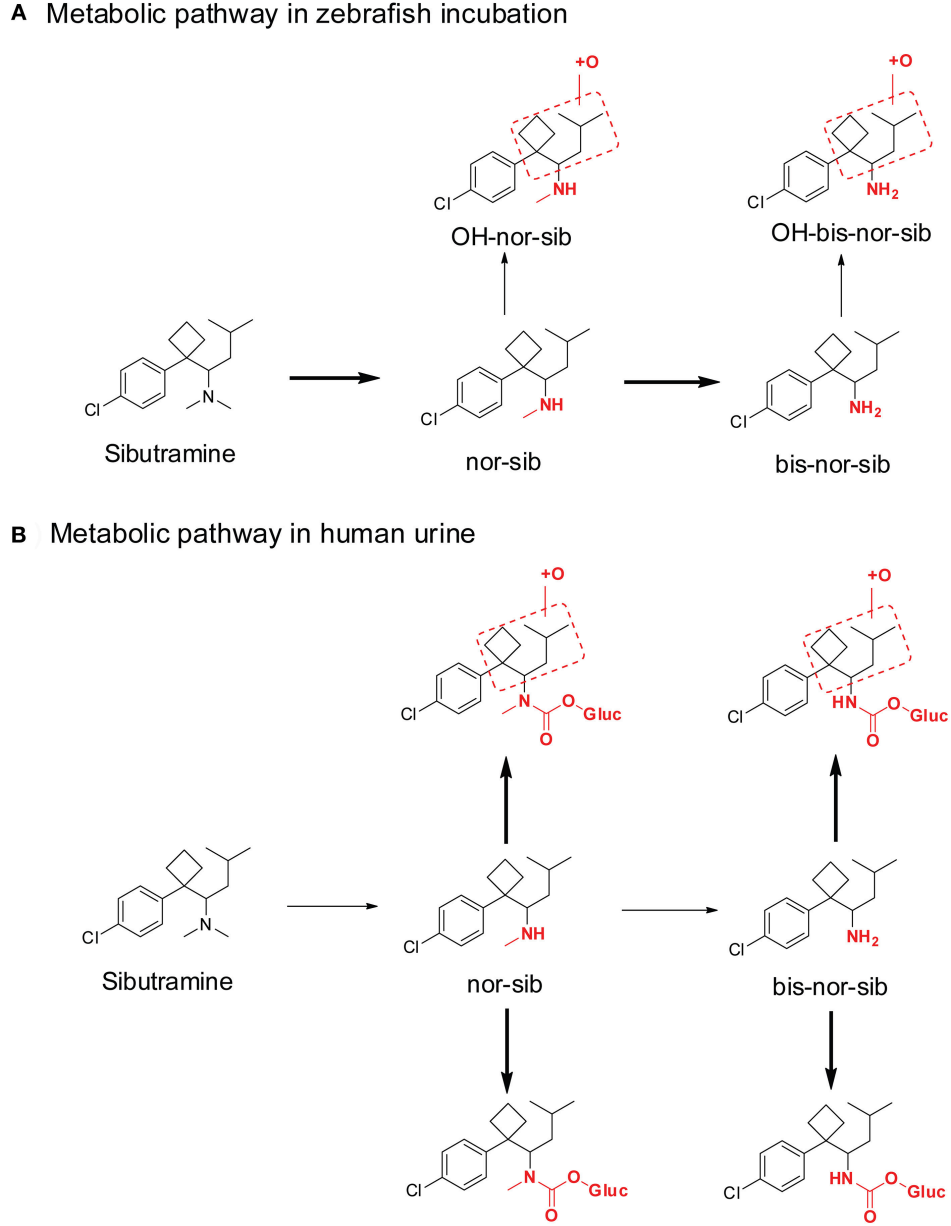
Metabolic pathway of sibutramine after Zebrafish incubation **(A)** and in human urine **(B)**. Bold arrows denote major metabolic pathways and narrow arrows for minor pathways.

The similarities between adult zebrafish and human metabolism were evaluated on the generation of phase I metabolites of the sports doping agent—sibutramine (de Souza Anselmo et al., 2017). Adult zebrafish produce several sibutramine metabolites (Figure 6A), including demethylsibutramine (nor-sib), bi-demethylsibutramine (bis-nor-sib), hydroxylated nor-sib (OH-nor-sib1 and OH-nor-sib2), and hydroxylated bis-nor-sib (OH-bis-nor-sib1 and OH-bis-nor-sib2). These metabolites were identified in zebrafish culture solution samples after hydrolysis with β-glucuronidase. The authors claimed that the study demonstrated that adult zebrafish could absorb, oxidize, and excrete several metabolites in a manner similar to humans. However, although these metabolites were observed in human urine, the major metabolites were quite different. In zebrafish incubation samples, the top two metabolites were demethylsibutramine (nor-sib) and bi-demethylsibutramine (bis-nor-sib), but, these two metabolites were only minor metabolites in human urine (Figure 6B). Eight phase II carbamoyl glucuronides of nor-sib and bis-nor-sib were the main metabolites in human urine (Link et al., 2006). Of note, the author did not hydrolyze the human urine sample with β-glucuronidase solution, so we cannot conclude whether nor-sib/bis-nor-sib or OH-nor-sib/OH-bis-nor-sib were the major human urine marker metabolites.

#### Advantages

Metabolism studies are convenient and feasible for laboratories with an available zebrafish culture platform. In such a setting, zebrafish culture is routine and cost-efficient, and it is easy to maintain and to train new staff. The zebrafish can produce many human-like phase I oxidative and reductive metabolites, and it may be possible to generate a large metabolite mass to allow isolation and structure elucidation.

#### Disadvantages

Typical forensic and clinical laboratories do not have experience with the zebrafish culture platform, and drug metabolism investigations take several days before harvesting the excretion sample in the culture tank. Species differences between zebrafish and human metabolism may limit expansion of the zebrafish cultural model.

### Fungus *C. elegans* Incubation

The use of microorganisms, and particularly fungus *C. elegans* as models to study human metabolism is well established and provides another approach to producing SC metabolites (Asha and Vidyavathi, 2009; Murphy, 2015). A review on *C. elegans* metabolism reported that the fungus has some similarities with human metabolism for various drugs (Asha and Vidyavathi, 2009). This cost-efficient system is capable of producing large quantities of metabolites. In addition, the fungus culture is easy to grow and can be transferred to new agar plates with ease (Choudhary et al., 2007).

Watanabe et al. made significant contributions on investigating the metabolism of SCs with the *C. elegans* model, and evaluated the similarity of metabolites generated in this incubation system with those observed in human metabolism (Watanabe et al., 2016, 2017, 2018a,b). SC metabolites for JWH-018, AM2201, JWH-073, PB-22, 5F-PB-22, XLR-11, and UR-144 were evaluated in the *C. elegans* incubation model. Some of the major phase I human metabolites of previously investigated SCs were documented, although this model was less effective in producing phase II human metabolites. The authors proposed that the fungus *C. elegans* is a complementary model to study human metabolism of novel SC and can generate sufficient SC metabolites for definitive structure elucidation.

**Figure 7:**
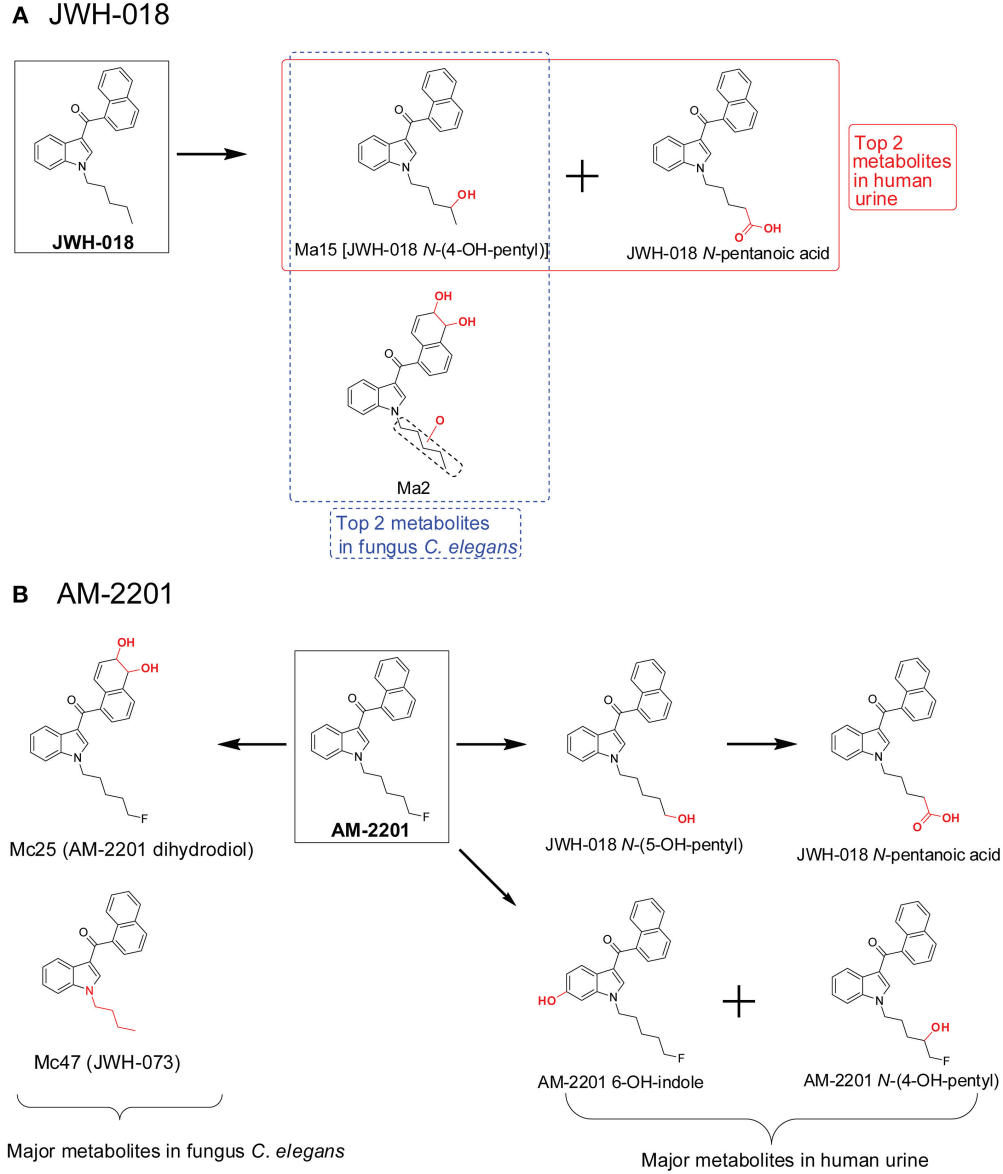
Metabolic pathway of JWH-018 **(A)** and AM-2201 **(B)** after fungus *Cunninghamella elegans* incubation vs. human urine.

When comparing *C. elegans* SC metabolites to human SC metabolites, there are similarities for some SCs but there were inconsistencies as well. For JWH-018 (Figure 7A), JWH-018 *N*-(4-OH-pentyl) and JWH-018 *N*-pentanoic acid were the top two metabolites in human urine collected from individuals with SC intoxication (Diao and Huestis, 2017). In the *C. elegans* incubation system, JWH-018 *N*-(4-OH-pentyl) was also the most abundant metabolite, matching quite well with the human metabolites (Watanabe et al., 2016). But the other abundant human metabolite, JWH-018 *N*-pentanoic acid, was a minor metabolite in *C. elegans* incubation model. For AM-2201 (Figure 7B), the primary human urinary metabolites were JWH-018 *N-*(5-OH-pentyl), JWH-018 *N-*pentanoic acid, AM-2201 6-OH-indole, AM-2201 *N-*(4-OH-pentyl). Although these metabolites were detected in the *C. elegans* incubation model, they had low abundance (Watanabe et al., 2016). In the *C. elegans* incubation model, the top two metabolites were Mc25 (AM-2201 dihydrodiol) and Mc47 (JWH-073). This is most likely attributed to the lack or low activity of the enzyme responsible for oxidative defluorination, which phenomenon was also observed in HLM incubation system.

#### Advantages

The *C. elegans* incubation system for metabolism of drugs offers a convenient, low cost approach for laboratories with access to this fungus culture platform. *C. elegans* culture is easy to maintain and train new staff. *C. elegans* incubation can generate many human phase I metabolites via different metabolic pathways, such as hydroxylation, dihydrodiol formation, carboxylation, dehydrogenation, and ketone formation etc. In addition, it is a useful model for large scale metabolite preparation compared to HLM or human hepatocytes incubation. In some cases, metabolite reference standards are difficult to synthesize and for example, the exact position of hydroxyl groups on the indole or indazole may not be elucidated by high-resolution mass spectrometry alone. Therefore, *C. elegans* incubation is a good model to produce and isolate metabolites.

#### Disadvantages

Most forensic and clinical laboratories do not have a fungus *C. elegans* culture platform and related experience. Drug metabolism investigations in *C. elegans* culture may take several days before harvesting the incubation sample. Although *C. elegans* have some CYP450 enzymes similar to those found in humans, inconsistencies in metabolism occur, limiting the expansion of the fungus *C. elegans* model to predict human SC metabolism.

## SC Metabolic Patterns

A typical SC structure contains a principal core (with various side chains), a linker, and a secondary moiety (Figure 1). The linker refers to the bridge between the principal core, i.e., pentylindole, and the secondary moiety, i.e., naphthalene. The linker can be carbonyl, ester or amide among different SCs (Andersson et al., 2016; Carlier et al., 2018).

Fluorine-for-hydrogen replacement at the terminal carbon of pentyl in pentylindole/pentylindazole SC is a typical SC structural design, which generally enhanced potency (Gurney et al., 2014). Such analogs include JWH-018/AM2201, PB-22/5F-PB-22, UR-144/XLR-11, AKB-48/5F-AKB-48, THJ-018/THJ-2201, AB-PINACA/5F-ABPINACA, and MN-18/5F-MN-18 etc. Wohlfarth et al proposed that these SC pairs shared similar major metabolic pathway patterns (Wohlfarth et al., 2015). In general, SCs with pentyl side chains were preferentially metabolized on the pentyl chain, especially the penultimate and terminal carbons. SCs with a 5-fluoropentyl side chain were predominantly metabolized on the terminal carbon, yielding 5-OH-pentyl and subsequent pentanoic acids. However, novel SCs emerging onto the abused-drug market have more diverse structures and this metabolic pathway may not occur. Later SC generations metabolism also may be different when an alternative to the early carbonyl linkage is present.

Based on our SC metabolism experience and other published literature, we summarize SC metabolism patterns as follows.

**Figure 8:**
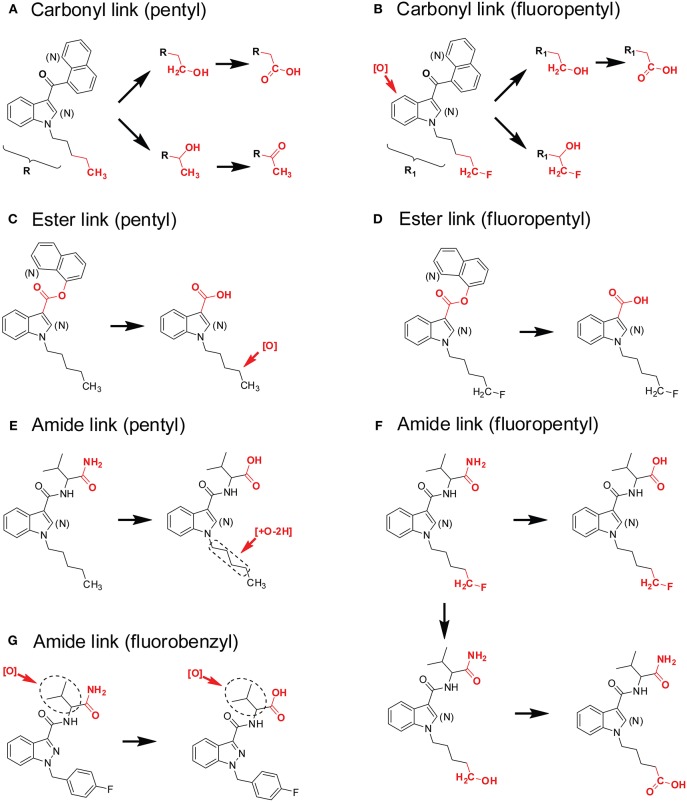
Metabolic patterns of SCs with different principal cores, linkers, and secondary moieties. Major metabolic sites were highlighted in red. **(A)** Carbonyl linker (pentyl), **(B)** Carbonyl linker (fluoropentyl), **(C)** Ester linker (pentyl), **(D)** Ester linker (fluoropentyl), **(E)** Amide linker (pentyl), **(F)** Amide linker (fluoropentyl), **(G)** Amide linker (fluorobenzyl).

1. Carbonyl linker (pentyl). This group includes SCs that contains a carbonyl linker, a principal core of a pentylindole or pentylindazole, and a secondary moiety of naphthalene or quinine (Figure 8A). The primary metabolic pathway for these SCs is hydroxylation at ω- (terminal) and ω-1- (penultimate) carbons of the pentyl side chain. Subsequent oxidation of ω-hydroxyl-pentyl and ω-1-hydroxyl-pentyl produces pentanoic acid and ω-1-carbonylated metabolites (Figure 8A). Besides pentyl chain containing SCs, these biotransformations also apply to pharmaceuticals that contains aliphatic side chains, such as sameridine and 3-*n*-butylphthalide (Sohlenius-Sternbeck et al., 2000; Diao et al., 2013a,b).
2. Carbonyl linker (fluoropentyl). This group includes SCs that have a carbonyl linker, a principal core of ω-fluoro-pentylindole or ω-fluoro-pentylindazole, and a secondary moiety of naphthalene or quinine (Figure 8B). Their predominant biotransformation is oxidative defluorination to ω-hydroxyl-pentyl SC and further oxidation to pentanoic acid; these two major metabolites were the same as those from corresponding SCs containing a pentyl side chain. Other major metabolites, mainly ω-1- and indole/indazole hydroxylated metabolites with retention of fluorine, are additional characteristic marker metabolites (Figure 8B).
3. Ester linker (pentyl). This group includes SCs that contain an ester linker, a principal core of pentylindole or pentylindazole, and a secondary moiety of naphthalene or quinine (Figure 8C). The most important metabolic pathway for these SCs was ester hydrolysis, rather than modification on the pentyl or fluoropentyl chain. The generated carboxylic acid was the single most abundant marker metabolite (Figure 8C). Although this carboxylic acid metabolite with a pentyl chain undergoes further ω-1-hydroxylation, the extent of this oxidation was much less compared with SCs with a carbonyl linker (Figure 8A).
4. Ester linker (fluoropentyl). These SCs contain an ester linker, a principal core of ω-fluoro-pentylindole or ω-fluoro-pentylindazole, and a secondary moiety of naphthalene or quinine (Figure 8D). Primary metabolism for these SCs also was ester hydrolysis, with little oxidative defluorination of the ω-fluoro-pentyl chain (Wohlfarth et al., 2014; Diao et al., 2017b).
5. Amide linker (pentyl). SCs that contain an amide linker, a principal core of a pentylindole or pentylindazole, and a secondary moiety of aminooxobutane comprise this group (Figure 8E). The primary metabolic pathway for these SCs is hydrolysis of the terminal amide to a carboxylic acid and further carbonylation, most likely on ω-1- carbon of the pentyl chain.
6. Amide linker (fluoropentyl). These SCs have an amide linker, a principal core of ω-fluoro-pentylindole or ω-fluoro-pentylindazole, and a secondary aminooxobutane moiety (Figure 8F). The major metabolic pathways for these SCs include not only hydrolysis of the terminal amide to carboxylic acid, but also oxidative defluorination of the ω-hydroxyl-pentyl SC and further oxidation to pentanoic acid.
7. Amide linker (fluorobenzyl). These SCs have an amide linker, a principal core of 4-fluoro-benzyl, and a secondary moiety of an aminooxobutane (Figure 8G). Hydrolysis of the terminal amide to a carboxylic acid is the most important metabolic pathway. Hydroxylation on the aminooxobutane occurred before and after hydrolysis.

## Identification of Specific SC Intake

Since human SC metabolism studies are rare and generally occur far after introduction of a new SC, one way to identify optimal urinary marker metabolites is performing metabolite profiling of authentic urine specimens (overdose emergency cases, driving under the influence of drugs cases, or when an individual found in possession of SCs). Paired blood and urine samples are difficult to obtain; they are highly valuable if available. In general, novel SCs are extensively metabolized primarily by human liver enzymes. Metabolites are mainly excreted in human urine, with parent SCs rarely detected in urine. Parent SC are detected in human blood and/or oral fluid if the sample is collected as close as possible to the time of intake. In addition, metabolites may be present in blood depending upon the dose and time after ingestion. However, caution is advised. Urinary metabolites may be present that derived from multiple SCs ingestion, while only one SC may be present in blood, confounding urine metabolite results.

We developed a strategy for characterizing SC metabolism and identifying suitable marker metabolites for new SC. The goal was to rapidly publish results to enable clinical and forensic toxicology laboratories to include target metabolites into SC screening and confirmation methods, and to identify optimal targets for reference manufacturers to synthesize as analytical standards.

We recommend the following SC metabolism workflow: (1) determine the SC's half-life in HLM to properly design human hepatocyte incubation; (2) incubate novel SC with human hepatocytes; (3) identify the most characteristic and abundant metabolites following hepatocytes incubation by HR-MS; (4) if possible, obtain authentic positive urine specimens and confirm marker metabolites. To offset the cost of hepatocytes, we recommend doing metabolism studies on 4–6 novel SCs at one time with one vial of human hepatocytes. The excellent quality data justifies human hepatocytes cost.

## Conclusion

SC abuse is a significant public health problem, resulting in many emergency department visits and fatalities. Despite illicit drug scheduling by governments, novel SCs are consistently introduced. To counter this growing challenge, global collaboration is critical. Rapid information sharing between government agencies and the scientific community is essential. Excellent examples of relevant efforts are the European Monitoring Centre for Drugs and Drug Addiction and the new United Nations Office on Drugs and Crime Early Warning Advisory Toxicology Portal. Rapid publication of marker metabolites and availability of human urine specimens to verify these markers for monitoring also are required.

Major hurdles are the cost of HR-MS for non-targeted urine SC screening, and the time and skills required to determine optimal SC marker metabolites from the highly complex HR-MS data obtained after injection of the SC human hepatocyte incubations (Pasin et al., 2017). Also, the size of the data generated in non-targeted SC screening techniques is immense, necessitating development of efficient and accurate data mining techniques. It is clear that SC urinary metabolites do not produce positive cannabinoid immunoassay tests; SC metabolite screening and confirmation assays need to be constantly updated to identify emerging SC intake. Collaboration between forensic toxicology laboratories and legitimate suppliers of analytical standards may result in better preparation and a timelier response to future SC outbreaks. Increased recognition and reporting by clinicians and public health personnel may aid federal and state regulatory efforts in combating this ongoing SC epidemic. It is important for clinicians and treatment personnel to stay abreast of local trends and, when necessary, partner with pharmacists, law enforcement, toxicologists, and mental health providers to discuss strategies for addressing SC intake and their resulting toxicities.

With global collaboration and communication, we can educate the public and improve our response to the introduction of novel SC. A positive trend is the recent reduction in new SC introduced per year, perhaps a result of global collaboration, especially the involvement of the Chinese government. However, as governments tighten scheduling laws, more complicated or uncommon principal cores or secondary substructures may emerge onto the market. The need for forensic toxicologists to identify the optimal target metabolites for these new SC and to investigate new metabolic patterns is likely to continue into the foreseeable future.

## Author Contributions

All authors listed have made a substantial, direct and intellectual contribution to the work, and approved it for publication.

### Conflict of Interest Statement

The authors declare that the research was conducted in the absence of any commercial or financial relationships that could be construed as a potential conflict of interest.
